# Effects of curcuminoids on allergic rhinitis diseases: a systematic review and meta-analysis of animal and human studies

**DOI:** 10.1186/s13643-026-03225-7

**Published:** 2026-06-09

**Authors:** Ratchanon Inpan, Kornvipa Settakorn, Nutthiya Hanprasertpong, Mingkwan Na Takuathung, Nut Koonrungsesomboon

**Affiliations:** 1 Department of Pharmacology, Faculty of Medicine, Chiang Mai University, Chiang Mai, 50200 Thailand; 2 Clinical Research Center for Food and Herbal Product Trials and Development (CR-FAH), Faculty of Medicine, Chiang Mai University, Chiang Mai, 50200 Thailand; 3 Office of Research Administration, Chiang Mai University, Chiang Mai, 50200 Thailand

**Keywords:** Allergic rhinitis, Curcuminoids, Systematic review, Meta-analysis

## Abstract

**Background:**

Allergic rhinitis is a chronic inflammatory condition triggered by allergens. Curcuminoids, known for their anti-inflammatory and immunomodulatory properties, show potential as alternative treatments for allergic rhinitis. This systematic review and meta-analysis evaluated the efficacy of curcuminoids in managing allergic rhinitis symptoms and related biological markers in both animal and human studies.

**Methods:**

A systematic search of PubMed, Embase, Scopus, and Cochrane Library was conducted to identify eligible animal and human studies. Data extraction included allergic rhinitis symptom scores (rubbing, sneezing, itching, nasal congestion, and runny nose) and related biological markers (i.e., IL-4 and IgE). The risk of bias was assessed using SYRCLE’s tool for animal studies and RoB2 for human studies. Meta-analyses employed a random-effects model and reported standardized mean difference (SMD) with 95% confidence intervals (CIs). Certainty of evidence was evaluated with the GRADE approach.

**Results:**

Seven animal studies (*n* = 189) and three human studies (*n* = 371) were included in the meta-analysis. Animal studies showed significant reductions in two symptom scores and one related biological marker: rubbing (SMD = − 2.2013, 95% CI − 3.8027 to − 0.5998, *p* = 0.0071); sneezing (SMD = − 2.3844, 95% CI − 3.6940 to − 1.0747, *p* = 0.0004); and IL-4 (SMD = − 0.9416, 95% CI − 1.8130 to − 0.0702, *p* = 0.0342). Symptom scores in humans showed a trend toward reduction, although the change was not statistically significant.

**Conclusions:**

Curcuminoids showed beneficial effects in animal models of allergic rhinitis, while current human evidence is limited and inconclusive. Further well-designed clinical trials are needed to clarify their therapeutic potential in humans.

**Systematic review registration:**

PROSPERO CRD42025640405.

**Supplementary Information:**

The online version contains supplementary material available at 10.1186/s13643-026-03225-7.

## Introduction

Allergic rhinitis is a chronic inflammatory condition of the nasal mucosa triggered by exposure to allergens such as house dust, dust mites, mold, and pollen [1]. It represents a significant global health issue, with its prevalence steadily increasing [2]. Over 500 million individuals worldwide are affected by allergic rhinitis, impacting both children and adults [3]. In the USA, an estimated 20–40 million people suffer from this condition, with prevalence rates ranging from 10 to 30% in adults and more than 40% in children [4].

The pathogenesis of allergic rhinitis begins when allergens enter the upper respiratory tract, activating T helper cells (CD4^+^). These cells differentiate into T helper 2 (TH2) cells, which release cytokines such as interleukin 4 (IL-4), interleukin 5 (IL-5), and interleukin 13 (IL-13). These cytokines stimulate the production of specific immunoglobulin E (IgE) from B cells, which binds to mast cells and basophils, the immune cells implicated in allergic reactions. Upon subsequent exposure to the same allergens, mast cells release histamine and other mediators, such as leukotrienes, prostaglandin D_2_ (PGD_2_), and tryptase, leading to the clinical manifestations of allergic rhinitis. The late-phase response follows hours later and triggers sustained inflammation, with eosinophils, neutrophils, T cells, and monocytes infiltrating the nasal mucosa. Eosinophils play a key role by releasing toxic granules major basic protein (MBP), eosinophil peroxidase (EPO), and eosinophil-derived neurotoxin (EDN), damaging the nasal epithelium and worsening inflammation. IL-5 promotes eosinophil activation, while interleukin 1 beta (IL-1β), tumor necrosis factor-alpha (TNF-α), and interleukin 6 (IL-6) further drive tissue damage, leading to chronic inflammation and persistent symptoms, including sneezing, itching, and nasal congestion [1].

Turmeric, scientifically known as *Curcuma longa* L., belongs to the *Zingiberaceae* family. The primary bioactive compounds in turmeric are curcumin, demethoxycurcumin, bisdemethoxycurcumin, and tetrahydrocurcumin, collectively known as curcuminoids, which are classified as polyphenolic compounds [5, 6]. These compounds are considered safe for oral consumption [7]. Extensive research on turmeric extract has demonstrated its diverse bioactive properties, including antioxidant, anti-inflammatory, neuroprotective, anticancer, hepatoprotective, and cardioprotective effects [8]. Furthermore, the effects of curcumin on allergic responses in mast cells have been studied in vitro. It inhibited histamine and β-hexosaminidase release, reduced reactive oxygen species (ROS) production, downregulated Fc epsilon receptor I (FcεRI) expression, and suppressed IL-4 and IL-13 activities [9]. Additionally, curcumin blocked protein kinase C delta (PKC-δ) translocation, suggesting its potential to alleviate IgE-induced allergic responses by reducing allergic mediator release [10]. This evidence indicates that curcuminoids may potentially improve allergic rhinitis symptoms and regulate related biological markers in animals and humans. Therefore, this research aims to systematically review and conduct a meta-analysis of animal and human studies to evaluate the efficacy of curcuminoids in treating allergic rhinitis.

## Materials and methods

### Literature search

The protocol was developed a priori following the guidelines outlined in the Preferred Reporting Items for Systematic Reviews and Meta-Analyses Protocol (PRISMA-P) 2015 statement. It was also officially registered with the International Prospective Register of Systematic Reviews (PROSPERO) under registration number CRD42025640405. Additionally, the study received an exempt research determination from the Research Ethics Committee of the Faculty of Medicine, Chiang Mai University (No. EXEMPTION 0062/2025).

In July 2024, comprehensive electronic database searches were conducted from their inception across four databases, that is, PubMed, Embase, Scopus, and the Cochrane Library, without any language restrictions. Final update in December 2024, the search strategy employed a wide range of terms related to the study population (e.g., animal or patients with allergic rhinitis conditions) and the interventions of interest were grouped based on their shared curcuminoid content (e.g., curcuminoids, curcumin, diferuloylmethane, demethoxycurcumin, bisdemethoxycurcumin, tetramethylcurcumin, curcuma, *Curcuma longa*, or turmeric). These two domains (i.e., study population and interventions) were combined using the “AND” operator to identify eligible studies across all databases as showed in Supplementary Table 1. Furthermore, a manual search of reference lists from relevant articles was performed by two independent reviewers to ensure the inclusion of potentially eligible studies not captured through electronic searches.

### Inclusion and exclusion criteria

Research articles examining changes in markers related to allergic rhinitis following curcuminoid interventions underwent eligibility assessment based on their titles and abstracts. The inclusion criteria specifically targeted studies involving animals or humans and were limited to randomized controlled trials (RCTs) comparing curcuminoids with a control. No restrictions were applied to the allergic rhinitis-related parameters assessed. Articles that did not meet these criteria, such as those without a control group, were excluded. Additionally, review articles, case reports, expert opinions, conference proceedings, and book chapters were excluded from the analysis.

### Data extractions

In this study, the data extraction process involved gathering the following elements: (1) the name of the first author; (2) the journal title and year of publication; (3) the study’s geographical location or study site (only in human studies); (4) demographic information such as participants or animals’ gender and age; (5) details of the intervention, including dosage and derivatives; (6) the intervention’s duration; (7) the number of participants or animals in each group; and (8) outcome measures related to allergic rhinitis, including symptom scores (rubbing score, sneezing score itching score, nasal congestion score, and runny nose score) and related biological markers (IL-4, IgE, and eosinophil count). Two reviewers carried out the data extraction independently, and any discrepancies were resolved through discussion and agreement. If the original publications lacked clarity, efforts were made to contact the corresponding authors via email for further information or clarification. For articles presenting data only in graphical format, WebPlotDigitizer was used to assist in extracting and calculating the data.

During the data extraction process, the focus was on collecting values that indicated changes from the baseline (or any necessary data for calculating such changes) for the specified outcomes, along with their corresponding standard deviations (SD). In cases where research studies used multiple dosages of curcuminoids but compared them to a single control group, the dosages from all curcuminoid groups were combined into one group for comparison with the control. However, in studies that examined multiple curcuminoid dosages and each dosage was paired with its respective control group, there was no requirement to calculate an average of the curcuminoid dosages.

### Risk of bias assessment

The risk of bias in animal studies was assessed using the Systematic review Centre for Laboratory Animal Experimentation (SYRCLE)’s tool composed of 10 entries across six domains: (1) selection bias, (2) performance bias, (3) detection bias, (4) attrition bias, (5) reporting bias, and (6) other biases. The risk of bias in RCTs was assessed using the Cochrane risk-of-bias tool for randomized trials (RoB2). The evaluation covered five domains to assess the potential for bias: (1) bias resulting from the randomization process, (2) bias due to deviations from the intended interventions, (3) bias from missing outcome data, (4) bias in the measurement of outcomes, and (5) bias in the selection of reported results. Each study, both in animal and human studies,was classified as having a low risk of bias, a high risk of bias, or some concerns about bias.

### Certainty of evidence assessment

The certainty of evidence in the study was assessed using the Grading of Recommendations Assessment, Development, and Evaluation (GRADE) approach, which included two categories of domains: (1) domains that could potentially lower the certainty in the evidence (i.e., risk of bias, inconsistency, indirectness, imprecision, and publication bias); and (2) domains that could increase the certainty in the evidence (i.e., large magnitude of effect, dose–response gradient, and the impact of plausible residual confounding). The certainty of the evidence was categorized into four levels: high, moderate, low, or very low.

### Statistical analyses

Meta-analyses were performed using the “meta” package in R Studio version 2024.12.0 (Posit Software, PBC, Boston, MA), build 467. A random-effects meta-analysis was conducted using the restricted maximum-likelihood method to calculate primary effect estimates, with statistical significance defined as a *p*-value < 0.05. Descriptive statistics for quantitative variables included the mean and SD. In animal studies, outcome variables were assessed by comparing post-treatment values between the curcuminoid intervention group and the control group. In contrast, human studies evaluated outcome variables by comparing the mean difference (post-treatment minus baseline values) between the intervention and control groups. Effect sizes were summarized using the standardized mean difference (SMD) and its corresponding 95% confidence intervals (95% CIs). Heterogeneity was evaluated using Cochran’s Q test and the *I*^2^ test. Forest plots and funnel plots were created with the “meta” package version 8.0–1, and a summary forest plot was produced using the “forestplot” package version 3.1.6. To explore potential small-study effects and identify publication bias, Egger’s test was employed to check for funnel plot asymmetry. Additionally, a sensitivity analysis was conducted using various estimation models, including the Dersimonian-Laird, Paule-Mandel, maximum-likelihood, Hunter-Schmidt, Sidik-Jonkman, Hedges, and empirical Bayes methods, to determine the most suitable random-effects model component. Studies identified as having a high risk of bias were excluded from the sensitivity analysis.

In cases where mean and SD values were not available, conversions from alternative metrics, such as median and interquartile range (IQR), were performed for each outcome. When SD values were missing, imputation methods were applied, or SD values were calculated from standard error (SE) or 95% confidence intervals (CIs), where applicable. Occasionally, a correlation coefficient (*r*) was used to estimate the SD of change from the baseline. If the necessary data to compute specific correlation coefficients for each outcome were unavailable, a default value of 0.5 was used.

To explore potential sources of variation by considering participant covariate factors or intervention characteristics, subgroup analyses were conducted alongside the primary analysis. Routes of administration were categorized as oral, sublingual, and steam bath. In addition, meta-regression analyses were performed to examine the effect of intervention duration. Subgroup analyses were performed only when a specific meta-analysis included at least three datasets to enable estimation of between-study heterogeneity, although power remains limited [11], while meta-regression analyses required at least five datasets to ensure sufficient degrees of freedom for the model to estimate the relationship between covariates and the effect size reliably [12].

## Results

### Literature search results

**Fig. 1:**
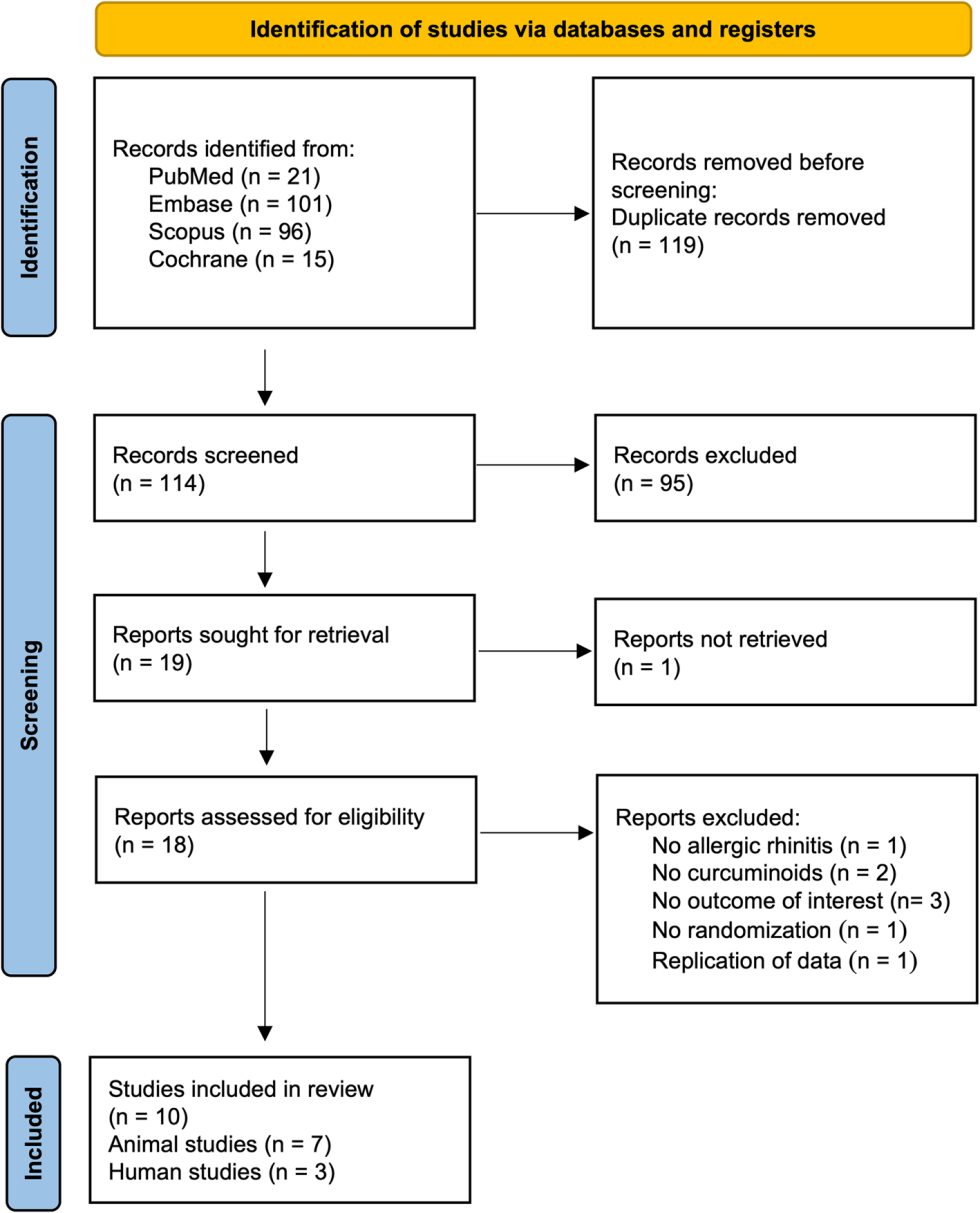
PRISMA flow diagram

Out of an initial pool of 233 articles, 119 were excluded due to duplication. The remaining 114 articles were screened based on their titles and abstracts, after which 18 articles were selected for full-text assessment. A total of 10 articles (animal studies, *n* = 7; human studies, *n* = 3) were determined to meet the eligibility criteria (Fig. 1 and Supplementary Table 2).

### Study characteristics and risk of bias assessment

A total of 10 articles were included in the analysis, all of which examined outcomes related to allergic rhinitis. The types of animals used were mice (*n* = 5; 157 mice), which had the largest population, followed by guinea pigs (*n* = 1; 18 guinea pigs) and rats (*n* = 1; 14 rats). For human studies, all were conducted in Asia: China (*n* = 1; 241 participants); Thailand (*n* = 1; 64 participants); and India (*n* = 1; 66 participants). The duration of the interventions varied, ranging from 3 to 60 days in animals, while in humans, the duration ranged from 28 to 84 days (Tables 1, and 2).

**Table 1:** Study characteristics in animals

Study	Animal	Intervention	Control	Induction	Treatment duration	Outcome	Ref
Thakare, 2013	Pigs	100 and 200 mg/kg curcumin (oral administration)	Saline	100 μg/animal ovalbumin and 5 mg/animal aluminum hydroxide	3 days	Rubbing, sneezing, IL-4, and IgE	[13]
Zhang, 2015	Mice	100 and 200 mg/kg curcumin (oral administration)	Saline	40 μg/kg ovalbumin and 40 mg/kg aluminum hydroxide	3 days	Rubbing, sneezing, and eosinophil cell count	[14]
Acar, 2016	Rats	4 mg curcumin (oral administration)	Distilled water	100 μg/animal ovalbumin and 5 mg/animal aluminum hydroxide	7 days	Rubbing and sneezing	[15]
Fu, 2018	Mice	50, 100 and 200 mg/kg bisdemethoxycurcumin (oral administration)	Distilled water	50 μg/animal ovalbumin and 2 mg/animal aluminum hydroxide	7 days	Rubbing and IgE	[16]
Shahgordi, 2020	Mice	5 and 10 μg curcumin and polylactic co-glycolic acid curcumin nanoparticles (sublingual administration)	Phosphate-buffered saline	10 μg/animal ovalbumin and 2 mg/animal aluminum hydroxide	60 days	Eosinophil cell count, and IL-4	[17]
Mo, 2021	Mice	5 mg tetramethylcurcumin (oral administration)	Saline	100 μg/animal ovalbumin in 0.1 ml aluminum hydroxide	7 days	Sneezing, IL-4, and IgE	[18]
Ansari, 2024	Mice	1.25 and 2.5 μg curcumin and nanofiber curcumin (Sublingual administration)	Phosphate-buffered saline	10 μg/animal ovalbumin and 2 mg/animal aluminum hydroxide	60 days	Eosinophil cell count, IL-4, and IgE	[19]

**Table 2:** Study characteristics in humans

Study	Location	Intervention	Control	Treatment duration	Outcome	Ref
Wu, 2016	China	500 mg/d curcumin	Placebo	60 days	Itching, nasal congestion, runny nose, and sneezing	[20]
Tungsukruthai, 2018	Thailand	75 g *Curcuma longa* in herbal steam bath	Steam bath	28 days	Itching, nasal congestion, runny nose, and sneezing	[21]
Mamatha, 2024	India	500 mg/d curcumin	Placebo	84 days	Itching, nasal congestion, runny nose, and sneezing	[22]

Among the animal studies considered, the majority of them (*n* = 5) were identified as having some concern regarding the risk of bias, whereas a smaller portion (*n* = 2) was categorized as having a high risk of bias, primarily due to issues related to sequence generation (selection bias) and blinding (detection bias) domains (Supplementary Fig. 1). Among the human studies, two studies were categorized as having some concern regarding the risk of bias, and one study was classified as having a high risk of bias, primarily due to issues related to bias due missing outcome data domain (Supplementary Fig. 2).

### Effect of curcuminoids on allergic rhinitis parameters

#### Animals

**Fig. 2:**
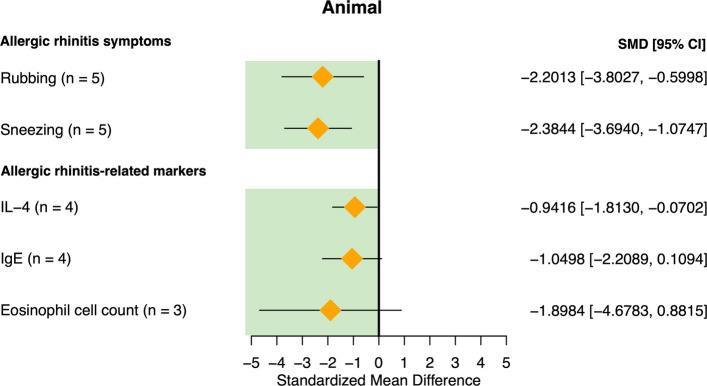
Forest plot summary of allergic rhinitis symptoms and allergic rhinitis-related biological markers standardized mean difference following the curcuminoid interventions vs. control in animals

In allergic rhinitis symptoms, rubbing presented a significant reduction (SMD = − 2.2013; 95% CI − 3.8027 to − 0.5998, *p*-value = 0.0071), and sneezing was significantly reduced (SMD = − 2.3844; 95% CI − 3.6940 to − 1.0747, *p*-value = 0.0004). Regarding allergic rhinitis-related biological markers, IL-4 levels significantly decreased following curcuminoid interventions (SMD = − 0.9416; 95% CI − 1.8130 to − 0.0702, *p*-value = 0.0342). However, no significant changes were observed, but there was a trend toward a reduction in ovalbumin-specific IgE and eosinophil cell counts in the tissue compared to the control group (Fig. 2). Funnel plot assessment indicated mixed evidence of symmetry across animal study outcomes. Egger’s test suggested potential small-study effects for rubbing (*p*-value = 0.0167), while results for sneezing were borderline (*p*-value = 0.0502). No significant asymmetry was detected for IL-4 (*p*-value = 0.3331), IgE (*p*-value = 0.0867), and eosinophil count (*p*-value = 0.4303). These findings are presented in Supplementary Figs. 3–7.

#### Humans

In human studies, only three randomized controlled trials have evaluated curcuminoid-related interventions in allergic rhinitis, exhibiting substantial heterogeneity in intervention type, formulation, administration route, study population, and outcome measures, thereby limiting the feasibility and robustness of pooled quantitative analyses. A large double-blind trial by Wu [20] demonstrated that oral curcumin significantly improved nasal symptoms and nasal airflow resistance and modulated systemic inflammatory markers, including reductions in IL-4, interleukin 8 (IL-8), and TNF-α and an increase in interleukin 10 (IL-10). In contrast, a smaller single-blind trial conducted in Thailand by Tungsukruthai [21] reported no curcuminoid-specific benefit of an herbal steam bath beyond non-specific symptom improvement. More recently, a double-blind placebo-controlled trial by Mamatha [22] showed that an oral curcumin co-delivery formulation significantly improved symptom scores, quality of life, and selected immune biomarkers significantly reduced pro-inflammatory cytokines, IL-1β, IL-6, TNF-α, CD4^+^, and CD4^+^/cytotoxic T lymphocytes (CD8^+^) ratio compared with placebo.

Among the three available studies, two used oral administration and one used steam bath administration, which represent fundamentally different mechanisms of action, reflecting systemic versus local/topical exposure, respectively. Pooling these routes in a single meta-analysis could therefore introduce substantial clinical heterogeneity and obscure true intervention effects. Accordingly, quantitative synthesis was restricted to studies using oral administration. The meta-analysis showed no significant changes in any symptom parameters following curcuminoid interventions compared to the control group (Supplementary Figs. 8–11).

#### Sensitivity analysis

A sensitivity analysis using various estimation models indicated results consistent with the main analysis for most parameters, except for IgE, which significantly reduced in animal studies with maximum-likelihood and Hunter-Schmidt estimators, and eosinophil count, which reduced with DerSimonian-Laird and Hunter-Schmidt estimators. Additionally, a sensitivity analysis excluding studies with a high risk of bias did not impact the results for most parameters, except for IL-4 in animal studies, which was no longer significantly reduced, and itching in humans, which became significantly reduced (Supplementary Table 3).

#### Subgroup analysis and meta-regression analysis

Most analyses in animal studies revealed considerable heterogeneity (*I*^2^ = 75–100%), including rubbing (88.1%), sneezing (80.6%), IgE (74.6%), and eosinophil counts (89.9%), whereas IL-4 exhibited substantial heterogeneity (*I*^2^ = 66.3%). To address this, subgroup analyses were carried out to explore potential sources of heterogeneity and assess the intervention’s impact on parameter variations within each subgroup. Following the subgroup analyses, heterogeneity remained high for most parameters. However, *I*^2^ was 0% for IL-4 with oral administration, and for both IL-4 and eosinophil cell counts with sublingual administration in animals (Supplementary Table 4). Furthermore, in the oral administration subgroup, curcuminoids significantly reduced all parameters compared to the control group, except for eosinophil cell count, for which there were insufficient studies for analysis. In contrast, the sublingual administration subgroup, curcuminoids significantly decreased eosinophil levels compared to the control group. However, the number of human studies was insufficient for subgroup analyses.

Meta-regression of the intervention duration on allergic rhinitis symptoms in animals found a non-significant association (rubbing, estimate = 0.1264, *p*-value = 0.7890; sneezing, estimate = − 0.1461, *p*-value = 0.7040) as represented in Supplementary Figs. 12–13.

### GRADE certainty of evidence assessment

The certainty of evidence for most parameters in humans was low, including itching, nasal congestion, runny nose, and sneezing, which were downgraded due to the inconsistency domain (Supplementary Table 5).

## Discussion

To the best of our knowledge, this study is the first systematic review and meta-analysis that was specifically designed to evaluate the effects of curcuminoids on allergic rhinitis. Our analysis found that curcuminoid intervention can alleviate allergic rhinitis symptoms in animal models, including rubbing and sneezing, by targeting key signaling markers such as IL-4 and IgE, which are closely associated with allergic inflammation. However, evidence from human studies remains limited. Our findings suggest a potential therapeutic role of curcuminoids in allergic rhinitis, while highlighting that current clinical evidence is insufficient and inconclusive, underscoring the need for further well-designed human studies.

Our systematic review and meta-analysis in animal models indicated that curcuminoids significantly decreased allergic rhinitis symptoms, such as rubbing and sneezing, compared to the control group. This improvement was associated with a reduction in allergic rhinitis-related biological markers, including IL-4, IgE, and eosinophil cells. Notably, curcuminoids have demonstrated strong regulatory effects on IL-4 in both in vitro and in vivo studies. They inhibited IL-4 production in house dust mite-stimulated peripheral blood mononuclear cells and suppressed IL-4-mediated TH2 immune responses in asthmatic mice, leading to reduced lung eosinophil infiltration [23]. In chronic asthma models, curcumin’s anti-allergic effects were associated with the inhibition of IL-4 and IL-5 production while promoting interferon gamma (IFN-γ) to restore the TH1/TH2 balance without triggering excessive pro-inflammatory responses. These findings emphasize curcuminoids’ potential to modulate IL-4-driven allergic responses [24]. Additionally, a novel curcumin delivery system enhanced its therapeutic efficacy by suppressing the activation of CD4^+^ cells and reducing IgE production [25].

Moreover, oral curcuminoid treatments effectively reduce IL-4 levels and slightly decrease IgE levels, alleviating early-phase allergic rhinitis symptoms. During the early phase, allergen exposure stimulates naive CD4^+^ cells to differentiate into TH2 cells and release IL-4, stimulating B cells to produce allergen-specific IgE, which binds to FcεRI on the surface of mast cells and basophils, sensitizing them for future exposures [26].

Eosinophil levels decreased slightly but not significantly with curcuminoid intervention, suggesting that curcuminoids may have limited effects on the late phase of allergic rhinitis. Eosinophils, recruited to the nasal mucosa through IL-4 and other TH2 cytokines such as IL-5, play a central role in promoting allergic inflammation during the late phase of allergic rhinitis [27]. Moreover, animal studies modeling allergic rhinitis using ovalbumin primarily simulate the early phase of the allergic response. As a result, curcuminoids show greater effects on early-phase biological markers, such as IL-4 and IgE, than on eosinophils, a late-phase marker [1]. The reduction of IL-4 and IgE decreases mast cell degranulation, which in turn limits eosinophil recruitment, explaining the minimal change in eosinophil levels.

However, sublingual administration of curcuminoids significantly reduced eosinophil activity, potentially due to the use of nanoparticle formulations that enhance curcuminoid bioavailability [28], thereby improving its effect on the late phase of allergic rhinitis. In contrast, oral treatments did not significantly reduce eosinophil levels, which may reflect limited gastrointestinal absorption of conventional curcuminoids rather than an absence of pharmacological activity. This distinction suggests that comparatively weaker results in animal studies may be driven, at least in part, by route of administration constraints rather than differences in intrinsic pharmacological properties. These findings also suggest that curcuminoids may exert relatively greater effects during the early phase of allergic rhinitis, where modulation of key mediators such as IL-4 and IgE contributes to initiation of the allergic response. In contrast, during the late phase characterized by eosinophil recruitment and sustained inflammation, limited systemic exposure associated with conventional oral formulations may be insufficient to effectively modulate these processes, resulting in less pronounced effects.

In human studies, the focus was on itching, nasal congestion, runny nose, and sneezing scores, which are assessed through the total nasal symptom score (TNSS) questionnaire for measuring allergic rhinitis symptoms [29]. Our study indicates that curcuminoids slightly decrease the symptoms of allergic rhinitis; however, there was no significant difference between the curcuminoid intervention and the control group, even when considering the route of administration as a subgroup. Importantly, the included human studies involved fundamentally different administration routes, such as oral capsules and herbal steam inhalation, which are not mechanistically comparable. Oral administration primarily exerts systemic immunomodulatory effects following absorption, whereas inhalation or steam-based approaches may act locally on the nasal mucosa with limited systemic exposure. These differences in pharmacokinetics and site of action may contribute to variability in outcomes and complicate direct comparisons across studies. Moreover, in human studies, allergic rhinitis is not directly stimulated with allergens as in animal models. As a result, cytokine levels may not reach peak levels during treatment with curcuminoids, leading to nonsignificant changes in cytokine levels and, consequently, minimal symptom improvement.

One key factor contributing to the more pronounced effect in animals is the controlled experimental environment. In animal studies, allergen sensitization is typically induced under highly controlled conditions (e.g., ovalbumin combined with aluminum hydroxide), alongside tightly regulated diet, genetic background, and immune responses, allowing for a more consistent and measurable therapeutic effect of curcuminoids [30]. In contrast, human studies involve natural and heterogeneous allergen exposure, including differences in allergen exposure, genetic predisposition, immune response, and environmental factors, which may dilute the observable effects of curcuminoids [31]. This fundamental difference between controlled sensitization in animal models and variable real-world exposure in humans likely represents a potential key factor underlying the reduced effects observed in clinical trials. Another important consideration is the dosage and bioavailability of curcuminoids. In animal studies, curcuminoids are often administered at higher, well-controlled doses with formulations that enhance their absorption and systemic availability. In humans, curcuminoids’ poor bioavailability, rapid metabolism, and limited absorption in the gastrointestinal tract may reduce their effectiveness, especially when taken orally [8].

This study has some limitations that should be taken into account. First, the number of included studies in both animal and human populations, was limited. In particular, data on blood parameters in human studies were insufficient for meta-analysis, restricting insights into early-phase immunological responses in humans. Second, despite subgroup analyses, heterogeneity remained substantial, and some outcomes lacked sufficient data for dosage-based subgrouping and meta-regression analysis. Accordingly, meta-regression did not demonstrate a significant association between intervention duration and symptom outcomes, suggesting no clear time-dependent effect, likely due to limited datasets and persistent heterogeneity. Third, the risk of bias remains a concern, as none of the included studies was assessed as low risk; most were classified as having unclear or some concerns, particularly regarding randomization and blinding. Furthermore, the reduction in IL-4 became non-significant after excluding high-risk studies, indicating limited robustness. This may partly be explained by the reduced sample size and consequent loss of statistical power, particularly given that the effect size remained comparable. The observed trend may still suggest a potential immunomodulatory effect. Consistent with these methodological limitations, the certainty of evidence assessment for all symptom outcomes in human studies was rated as low, indicating substantial uncertainty and the need for cautious interpretation. Lastly, most included studies evaluated short-term curcuminoid interventions, providing limited evidence on long-term efficacy and safety. Consequently, their role in sustained clinical use remains uncertain.

**Fig. 3:**
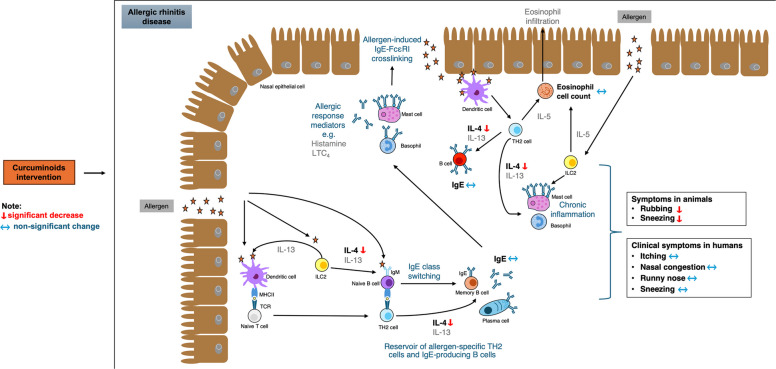
The potential pathway of curcuminoid intervention on allergic rhinitis parameters. This pathway has been modified from [1]. The parameters included in our analysis are highlighted in bold, colored text, while parameters that were insufficient for analysis are shown in gray. The red downward arrow represents parameters that showed significant decrease, whereas the blue left–right arrow represents parameters with non-significant change

Our systematic review and meta-analysis demonstrate that curcuminoid interventions may offer benefits for allergic rhinitis. Curcuminoids may exert their effects by modulating key immune responses at different stages of the allergic reaction. During the sensitization phase, curcuminoids significantly reduce IL-4, a cytokine that drives TH2 cell differentiation and stimulates B cells to produce allergen-specific IgE antibodies. By lowering IL-4 levels, curcuminoids show a trend toward suppressing IgE synthesis, thereby reducing mast cell sensitization and priming for future allergen exposure. This, in turn, limits mast cell degranulation and the release of inflammatory mediators such as histamine, leukotrienes, and prostaglandins in the early-phase reaction. In the late-phase reaction, which occurs hours later and is driven by eosinophilic inflammation, curcuminoids showed a mild but non-significant decrease in eosinophil levels in animal models. This suggests that while curcuminoids help regulate IL-4, they may not sufficiently suppress IL-5-mediated eosinophil recruitment, which plays a key role in prolonged nasal congestion and inflammation. However, symptoms such as rubbing and sneezing were reduced in animal models of allergic rhinitis, possibly due to IL-4 reduction, highlighting curcuminoids’ potential to address multiple factors in the allergic response. This promising effect in animal models suggests a tendency for similar benefits in humans. Although human demonstrated only modest and non-significant reductions in symptoms such as itching, nasal congestion, rhinorrhea, and sneezing, consistent improvements observed in animal models support the biological plausibility of curcuminoids in allergic rhinitis (Fig. 3). However, the limited and inconsistent availability of human biomarker data precludes definitive conclusions regarding clinical efficacy and mechanisms in humans.

Further well-designed, adequately powered clinical trials with standardized outcome measures, comprehensive biomarker profiling, and longer follow-up periods are required to clarify the therapeutic role of curcuminoids in allergic rhinitis, including long-term safety, durability of symptom control, recurrence after allergen re-exposure, and sustained quality-of-life effects. Although this review is predominantly informed by animal studies, it identifies consistent effect patterns across experimental models, strengthens inference beyond individual studies, and advances mechanistic insight to guide future clinical research. Nonetheless, these findings should be interpreted as supportive preclinical evidence rather than direct proof of clinical efficacy.

## Conclusion

This systematic review and meta-analysis suggests that curcuminoids may improve allergic rhinitis outcomes, with more consistent effects observed in preclinical models. Benefits appear more evident in early-phase immune responses (e.g., IL-4, IgE), whereas findings for late-phase inflammatory markers (e.g., eosinophils) are less consistent and frequently non-significant, which may limit their role as a standalone treatment for chronic inflammation. Evidence from human studies remains limited and heterogeneous, precluding definitive conclusions regarding clinical efficacy. Overall, the role of curcuminoids in allergic rhinitis remains uncertain, and further well-designed clinical trials with standardized outcomes and longer follow-up are needed to clarify their efficacy and clinical relevance.

## Supplementary Information

Supplementary Material 1.

## Data Availability

All data used to support the findings of this study are available from the corresponding author upon reasonable request.

## Abbreviations

CIs: Confidence intervals
CD8^+^: Cytotoxic T lymphocytes
EPO: Eosinophil peroxidase
EDN: Eosinophil-derived neurotoxin
FcεRI: Fc epsilon receptor I
IgA: Immunoglobulin A
IgG: Munoglobulin G
IgM: Immunoglobulin M
IFN-γ: Interferon gamma
IL-4: Interleukin 4
IL-5: Interleukin 5
IL-6: Interleukin 6
IL-8: Interleukin 8
IL-10: Interleukin 10
IL-13: Interleukin 13
IL-1β: Interleukin-1 beta
IQR: Interquartile range
MBP: Major basic protein
PGD2: Prostaglandin D2
PKC-δ: Protein kinase C delta
RCTs: Randomized controlled trials
ROS: Reactive oxygen species
IgE: Specific immunoglobulin E
SD: Standard deviation
SE: Standard error
SMD: Standardized mean difference
CD4^+^: T helper
TH1: T helper 1
TH2: T helper 2
GRADE: The Grading of Recommendations Assessment, Development, and Evaluation
PROSPERO: The International Prospective Register of Systematic Reviews
PRISMA-P: The Preferred Reporting Items for Systematic Reviews and Meta-Analyses Protocol
SYRCLE: The Systematic Review Centre for Laboratory Animal Experimentation
TNSS: Total nasal symptom score
TNF-α: Tumor necrosis factor alpha
