# Supplementary material for: Effects of curcuminoids on allergic rhinitis diseases: a systematic review and meta-analysis of animal and human studies
Source: Syst Rev. 2026 Jun 9;15:224. doi: 10.1186/s13643-026-03225-7 (PMC13471372; doi:10.1186/s13643-026-03225-7)
Supplement: Supplementary file 1 [file 13643_2026_3225_MOESM1_ESM.docx]

**Supplementary Information**

**Effects of curcuminoids on allergic rhinitis diseases: a systematic review and meta-analysis of animal and human studies**

Ratchanon Inpan^1,2,3^, Kornvipa Settakorn^1,2^, Nutthiya Hanprasertpong^1,2^, Mingkwan Na Takuathung^1,2^, Nut Koonrungsesomboon^1,2*^

^1^ Department of Pharmacology, Faculty of Medicine, Chiang Mai University, Chiang Mai 50200, Thailand

^2^ Clinical Research Center for Food and Herbal Product Trials and Development (CR-FAH), Faculty of Medicine, Chiang Mai University, Chiang Mai 50200, Thailand

^3^ Office of Research Administration, Chiang Mai University, Chiang Mai 50200, Thailand

**^*^Corresponding author** Nut Koonrungsesomboon, M.D., Ph.D., Department of Pharmacology, Faculty of Medicine, Chiang Mai University, Chiang Mai 50200, Thailand.

**List of all authors’ email addresses**

ratchanon.inpan@gmail.com and ratchanon.inpan@cmu.ac.th (R. Inpan),

stkornvipa@gmail.com and kornvipa.s@cmu.ac.th (K. Settakorn),

knutthiya@gmail.com (N. Hanprasertpong),

k_mingkwan@hotmail.com (M. Na Takuathung),

nkoonrung@gmail.com and nut.koonrung@cmu.ac.th. (N. Koonrungsesomboon).

**Supplementary Figure Legends**

**Supplementary Figure 1** SYRCLE’s risk of bias assessment in animal

**Supplementary Figure 2** ROB2’s risk of bias assessment in human

**Supplementary Figure 3** Forest plot and funnel plot of rubbing following the curcuminoids intervention vs. control in animal

**Supplementary Figure 4** Forest plot and funnel plot of sneezing following the curcuminoids intervention vs. control in animal

**Supplementary Figure 5** Forest plot and funnel plot of IL-4 following the curcuminoids intervention vs. control in animal

**Supplementary Figure 6** Forest plot and funnel plot of IgE following the curcuminoids intervention vs. control in animal

**Supplementary Figure 7** Forest plot and funnel plot of eosinophil cell count following the curcuminoids intervention vs. control in animal

**Supplementary Figure 8** Forest plot of itching following the curcuminoids intervention vs. control in human

**Supplementary Figure 9** Forest plot of nasal congestion following the curcuminoids intervention vs. control in human

**Supplementary Figure 10** Forest plot of runny nose following the curcuminoids intervention vs. control in human

**Supplementary Figure 11** Forest plot of sneezing following the curcuminoids intervention vs. control in human

**Supplementary Figure 12** Meta-regression of rubbing following the curcuminoids intervention vs. control in animal

**Supplementary Figure 13** Meta-regression of sneezing following the curcuminoids intervention vs. control in animal

**Supplementary Table Legends**

**Supplementary Table 1** Detailed search strategies used across databases

**Supplementary Table 2** List of full-text articles excluded after eligibility assessment

**Supplementary Table 3** Sensitivity analysis on allergic rhinitis markers following the curcuminoids intervention vs. control in animal and human

**Supplementary Table 4** Subgroup analysis on allergic rhinitis markers following the curcuminoids intervention vs. control in animal and human

**Supplementary Table 5** A summary of GRADE’s approach to rating the quality of evidence in human

## **Supplementary Table 1**

| **Domain** | **Database** | | | |
| --- | --- | --- | --- | --- |
|  | **Pubmed** | **Embase** | **Scopus** | **Cochrane** |
| ***Population*** | | | | |
| Allergic rhinitis OR Allergic rhinopathy OR Perennial Allergic Rhinitis OR Rhinitis OR Seasonal Allergic Rhinitis | 52,097 | 75,470 | 76,152 | 11,721 |
| ***Intervention*** | | | | |
| Curcuminoids OR Curcumin OR Diferuloylmethane OR Demethoxycurcumin OR Bisdemethoxycurcumin OR Tetramethylcurcumin OR Curcuma OR Curcuma longa OR Turmeric | 26,969 | 47,766 | 55,158 | 2,535 |
| ***Combination*** | | | | |
| ((Allergic rhinitis OR Allergic rhinopathy OR Perennial Allergic Rhinitis OR Rhinitis OR Seasonal Allergic Rhinitis) AND (Curcuminoids OR Curcumin OR Diferuloylmethane OR Demethoxycurcumin OR Bisdemethoxycurcumin OR Tetramethylcurcumin OR Curcuma OR Curcuma longa OR Turmeric)) | 21 | 101 | 96 | 15 |
| **Sum** | 233 | | | |
| **Duplication** | 119 | | | |
| **Remain** | 114 | | | |

## **Supplementary Table 2**

| **Author, year** | **Title** | **DOI** | **Reason** |
| --- | --- | --- | --- |
| Zhang, 2007 | The role of protein kinase C and activator protein-1 signal cascade in the regulation of expression of interleukin-5 in T lymphocyte of allergic rhinitis | NA | No outcome of interest |
| Bhaskaran, 2012 | Clinical evaluation of Amragandha haridra (*Curcuma amada* Roxb) in Pratisyaya W.S.R to allergic Rhinitis: A folklore claim | NA | No curcuminoids |
| Storka, 2015 | Safety, tolerability and pharmacokinetics of liposomal curcumin in healthy humans | 10.5414/CP202076 | No randomization |
| Altıntoprak, 2016 | Antioxidant activities of curcumin in allergic rhinitis | 10.1007/s00405-016-4076-4 | No outcome of interest |
| Kawasaki, 2017 | Effect of a combination of hot water extract of curcuma longa and curcumin on serum liver enzymes, inflammatory markers, and emotional states in healthy participants with moderately high body mass index - A randomized, double-blind, placebo-controlled clinical trial | NA | Reports not retrieved |
| Kim, 2020 | Activation of the Nrf2/HO-1 pathway by curcumin inhibits oxidative stress in human nasal fibroblasts exposed to urban particulate matter | 10.1186/s12906-020-02886-8 | No allergic rhinitis |
| Yu, 2020 | Overexpression of hypoxia-inducible factor 1α is associated with neutrophilic inflammation in chronic rhinosinusitis with nasal polyps | 10.1016/j.anl.2019.09.011 | No curcuminoids |
| Lee, 2023 | Safety evaluation of high bioavailability curcumin in Healthy Japanese Adults: A Randomized, placebo-controlled, double-blind, parallel-group comparison study | 10.31989/ffhd.v13i12.1207 | No outcome of interest |
| Mamatha, 2024 | Short-term influence of Immufen™ on mild allergic rhinitis: a randomized, double-blind, placebo-controlled study | 10.3389/falgy.2024.1390813 | Replication of data |

## **Supplementary Table 3**

| **Outcome** | **Analytic method** | **Number of studies** | **Number of animals/participants** | | **Heterogeneity** | | **Effect estimates** | | |
| --- | --- | --- | --- | --- | --- | --- | --- | --- | --- |
|  |  |  | **Intervention** | **Control** | **Chi^2^** | **I^2^ (%)** | **Standardized Mean Difference** | **95% CI** | **p-value** |
| *Animal* | |  |  |  |  |  |  |  |  |
| Rubbing | Main analysis | 5 | 77 | 37 | < 0.0001 | 88.1 | -2.2013 | -3.8027 to -0.5998 | 0.0071 |
|  | Fixed-effect estimate | 5 | 77 | 37 | - | - | -1.2705 | -1.7633 to -0.7777 | < 0.0001 |
|  | DerSimonian-Laird | 5 | 77 | 37 | < 0.0001 | 88.1 | -2.1957 | -3.7641 to -0.6273 | 0.0061 |
|  | Paule-Mandel | 5 | 77 | 37 | < 0.0001 | 88.1 | -2.1985 | -3.7831 to -0.6138 | 0.0065 |
|  | Maximum-likelihood | 5 | 77 | 37 | < 0.0001 | 88.1 | -2.1694 | -3.6049 to -0.7339 | 0.0031 |
|  | Hunter-Schmidt | 5 | 77 | 37 | < 0.0001 | 88.1 | -2.1414 | -3.4664 to -0.8164 | 0.0015 |
|  | Sidik-Jonkman | 5 | 77 | 37 | < 0.0001 | 88.1 | -2.1972 | -3.7745 to -0.6199 | 0.0063 |
|  | Hedges | 5 | 77 | 37 | < 0.0001 | 88.1 | -2.1950 | -3.7592 to -0.6307 | 0.0060 |
|  | Empirical Bayes | 5 | 77 | 37 | < 0.0001 | 88.1 | -2.1985 | -3.7831 to -0.6138 | 0.0065 |
|  | Excluded high risk of bias | 4 | 70 | 30 | < 0.0001 | 88.6 | -1.8399 | -3.5902 to -0.0896 | 0.0394 |
| Sneezing | Main analysis | 5 | 53 | 33 | 0.0004 | 80.6 | -2.3844 | -3.6940 to -1.0747 | 0.0004 |
|  | Fixed-effect estimate | 5 | 53 | 33 | - | - | -1.9652 | -2.5652 to -1.3651 | < 0.0001 |
|  | DerSimonian-Laird | 5 | 53 | 33 | 0.0004 | 80.6 | -2.3994 | -3.8115 to -0.9873 | 0.0009 |
|  | Paule-Mandel | 5 | 53 | 33 | 0.0004 | 80.6 | -2.3786 | -3.6544 to -1.1028 | 0.0003 |
|  | Maximum-likelihood | 5 | 53 | 33 | 0.0004 | 80.6 | -2.3603 | -3.5438 to -1.1767 | < 0.0001 |
|  | Hunter-Schmidt | 5 | 53 | 33 | 0.0004 | 80.6 | -2.3719 | -3.6114 to -1.1324 | 0.0002 |
|  | Sidik-Jonkman | 5 | 53 | 33 | 0.0004 | 80.6 | -2.3791 | -3.6575 to -1.1007 | 0.0003 |
|  | Hedges | 5 | 53 | 33 | 0.0004 | 80.6 | -2.3718 | -3.6106 to -1.1329 | 0.0002 |
|  | Empirical Bayes | 5 | 53 | 33 | 0.0004 | 80.6 | -2.3786 | -3.6544 to -1.1028 | 0.0003 |
|  | Excluded high risk of bias | 3 | 40 | 20 | 0.0002 | 88.1 | -2.1969 | -4.1713 to -0.2225 | 0.0292 |
| IL-4 | Main analysis | 4 | 82 | 44 | 0.0308 | 66.3 | -0.9416 | -1.8130 to -0.0702 | 0.0342 |
|  | Fixed-effect estimate | 4 | 82 | 44 | - | - | -0.6834 | -1.0728 to -0.2939 | 0.0006 |
|  | DerSimonian-Laird | 4 | 82 | 44 | 0.0308 | 66.3 | -0.9167 | -1.7085 to -0.1248 | 0.0233 |
|  | Paule-Mandel | 4 | 82 | 44 | 0.0308 | 66.3 | -0.9464 | -1.8354 to -0.0574 | 0.0369 |
|  | Maximum-likelihood | 4 | 82 | 44 | 0.0308 | 66.3 | -0.8840 | -1.5936 to -0.1744 | 0.0146 |
|  | Hunter-Schmidt | 4 | 82 | 44 | 0.0308 | 66.3 | -0.8447 | -1.4754 to -0.2141 | 0.0087 |
|  | Sidik-Jonkman | 4 | 82 | 44 | 0.0308 | 66.3 | -0.9498 | -1.8521 to -0.0476 | 0.0391 |
|  | Hedges | 4 | 82 | 44 | 0.0308 | 66.3 | -0.9475 | -1.8405 to -0.0544 | 0.0376 |
|  | Empirical Bayes | 4 | 82 | 44 | 0.0308 | 66.3 | -0.9464 | -1.8354 to -0.0574 | 0.0369 |
|  | Excluded high risk of bias | 3 | 76 | 38 | 0.0282 | 72.0 | -0.8102 | -1.8789 to 0.2586 | 0.1373 |
| IgE | Main analysis | 4 | 64 | 30 | 0.0081 | 74.6 | -1.0498 | -2.2089 to 0.1094 | 0.0759 |
|  | Fixed-effect estimate | 4 | 64 | 30 | - | - | -0.6531 | -1.1312 to -0.1749 | 0.0074 |
|  | DerSimonian-Laird | 4 | 64 | 30 | 0.0081 | 74.6 | -1.0110 | -2.0396 to 0.0177 | 0.0541 |
|  | Paule-Mandel | 4 | 64 | 30 | 0.0081 | 74.6 | -1.0631 | -2.2769 to 0.1506 | 0.0860 |
|  | Maximum-likelihood | 4 | 64 | 30 | 0.0081 | 74.6 | -0.9841 | -1.9399 to -0.0283 | 0.0436 |
|  | Hunter-Schmidt | 4 | 64 | 30 | 0.0081 | 74.6 | -0.9412 | -1.8005 to -0.0820 | 0.0318 |
|  | Sidik-Jonkman | 4 | 64 | 30 | 0.0081 | 74.6 | -1.0648 | -2.2860 to 0.1563 | 0.0874 |
|  | Hedges | 4 | 64 | 30 | 0.0081 | 74.6 | -1.0734 | -2.3334 to 0.1866 | 0.0950 |
|  | Empirical Bayes | 4 | 64 | 30 | 0.0081 | 74.6 | -1.0631 | -2.2769 to 0.1506 | 0.0860 |
|  | Excluded high risk of bias | 3 | 58 | 24 | 0.0633 | 63.8 | -0.6033 | -1.5095 to 0.3029 | 0.1920 |
| Eosinophil cell count | Main analysis | 3 | 78 | 39 | < 0.0001 | 89.9 | -1.8984 | -4.6783 to 0.8815 | 0.1807 |
|  | Fixed-effect estimate | 3 | 78 | 39 | - | - | -0.8633 | -1.2878 to -0.4389 | < 0.0001 |
|  | DerSimonian-Laird | 3 | 78 | 39 | < 0.0001 | 89.9 | -1.7161 | -3.4074 to -0.0247 | 0.0467 |
|  | Paule-Mandel | 3 | 78 | 39 | < 0.0001 | 89.9 | -1.9040 | -4.7514 to 0.9435 | 0.1900 |
|  | Maximum-likelihood | 3 | 78 | 39 | < 0.0001 | 89.9 | -1.8302 | -4.0297 to 0.3693 | 0.1029 |
|  | Hunter-Schmidt | 3 | 78 | 39 | < 0.0001 | 89.9 | -1.4848 | -2.6636 to -0.3060 | 0.0136 |
|  | Sidik-Jonkman | 3 | 78 | 39 | < 0.0001 | 89.9 | -1.9002 | -4.7017 to 0.9012 | 0.1837 |
|  | Hedges | 3 | 78 | 39 | < 0.0001 | 89.9 | -1.9086 | -4.8165 to 0.9993 | 0.1983 |
|  | Empirical Bayes | 3 | 78 | 39 | < 0.0001 | 89.9 | -1.9040 | -4.7514 to 0.9435 | 0.1900 |
|  | Excluded high risk of bias | 3 | 78 | 39 | < 0.0001 | 89.9 | -1.8984 | -4.6783 to 0.8815 | 0.1807 |
| *Human* |  |  |  |  |  |  |  |  |  |
| Itching | Main analysis | 2 | 162 | 145 | < 0.0001 | 99.2 | -2.2564 | -5.9677 to 1.4548 | 0.2334 |
|  | Fixed-effect estimate | 2 | 162 | 145 | - | - | -2.3990 | -2.7299 to -2.0682 | < 0.0001 |
|  | DerSimonian-Laird | 2 | 162 | 145 | < 0.0001 | 99.2 | -2.2564 | -5.9677 to 1.4548 | 0.2334 |
|  | Paule-Mandel | 2 | 162 | 145 | < 0.0001 | 99.2 | -2.2564 | -5.9677 to 1.4548 | 0.2334 |
|  | Maximum-likelihood | 2 | 162 | 145 | < 0.0001 | 99.2 | -2.2576 | -4.8818 to 0.3667 | 0.0918 |
|  | Hunter-Schmidt | 2 | 162 | 145 | < 0.0001 | 99.2 | -2.2576 | -4.8744 to 0.3592 | 0.0909 |
|  | Sidik-Jonkman | 2 | 162 | 145 | < 0.0001 | 99.2 | -2.2564 | -5.9533 to 1.4404 | 0.2316 |
|  | Hedges | 2 | 162 | 145 | < 0.0001 | 99.2 | -2.2564 | -5.9677 to 1.4548 | 0.2334 |
|  | Empirical Bayes | 2 | 162 | 145 | < 0.0001 | 99.2 | -2.2564 | -5.9677 to 1.4548 | 0.2334 |
|  | Excluded high risk of bias | 1 | 33 | 33 | - | - | - | - | - |
| Nasal congestion | Main analysis | 2 | 162 | 145 | < 0.0001 | 99.0 | -1.8341 | -5.0353 to 1.3671 | 0.2615 |
|  | Fixed-effect estimate | 2 | 162 | 145 | - | - | -2.1319 | -2.4409 to -1.8229 | < 0.0001 |
|  | DerSimonian-Laird | 2 | 162 | 145 | < 0.0001 | 99.0 | -1.8341 | -5.0353 to 1.3671 | 0.2615 |
|  | Paule-Mandel | 2 | 162 | 145 | < 0.0001 | 99.0 | -1.8341 | -5.0353 to 1.3671 | 0.2615 |
|  | Maximum-likelihood | 2 | 162 | 145 | < 0.0001 | 99.0 | -1.8370 | -4.1006 to 0.4266 | 0.1117 |
|  | Hunter-Schmidt | 2 | 162 | 145 | < 0.0001 | 99.0 | -1.8372 | -4.0628 to 0.3885 | 0.1057 |
|  | Sidik-Jonkman | 2 | 162 | 145 | < 0.0001 | 99.0 | -1.8341 | -5.0204 to 1.3522 | 0.2592 |
|  | Hedges | 2 | 162 | 145 | < 0.0001 | 99.0 | -1.8341 | -5.0353 to 1.3671 | 0.2615 |
|  | Empirical Bayes | 2 | 162 | 145 | < 0.0001 | 99.0 | -1.8341 | -5.0353 to 1.3671 | 0.2615 |
|  | Excluded high risk of bias | 1 | 33 | 33 | - | - | - | - | - |
| Runny nose | Main analysis | 2 | 162 | 145 | < 0.0001 | 99.5 | -2.8512 | -7.9418 to 2.2395 | 0.2723 |
|  | Fixed-effect estimate | 2 | 162 | 145 | - | - | -2.5171 | -2.8813 to -2.1530 | < 0.0001 |
|  | DerSimonian-Laird | 2 | 162 | 145 | < 0.0001 | 99.5 | -2.8512 | -7.9418 to 2.2395 | 0.2723 |
|  | Paule-Mandel | 2 | 162 | 145 | < 0.0001 | 99.5 | -2.8512 | -7.9418 to 2.2395 | 0.2723 |
|  | Maximum-likelihood | 2 | 162 | 145 | < 0.0001 | 99.5 | -2.8494 | -6.4490 to 0.7502 | 0.1208 |
|  | Hunter-Schmidt | 2 | 162 | 145 | < 0.0001 | 99.5 | -2.8493 | -6.4191 to 0.7204 | 0.1177 |
|  | Sidik-Jonkman | 2 | 162 | 145 | < 0.0001 | 99.5 | -2.8511 | -7.9288 to 2.2265 | 0.2711 |
|  | Hedges | 2 | 162 | 145 | < 0.0001 | 99.5 | -2.8512 | -7.9418 to 2.2395 | 0.2723 |
|  | Empirical Bayes | 2 | 162 | 145 | < 0.0001 | 99.5 | -2.8512 | -7.9418 to 2.2395 | 0.2723 |
|  | Excluded high risk of bias | 1 | 33 | 33 | - | - | - | - | - |
| Sneezing | Main analysis | 2 | 162 | 145 | < 0.0001 | 99.3 | -2.9045 | -7.3438 to 1.5347 | 0.1997 |
|  | Fixed-effect estimate | 2 | 162 | 145 | - | - | -2.7547 | -3.1165 to -2.3929 | < 0.0001 |
|  | DerSimonian-Laird | 2 | 162 | 145 | < 0.0001 | 99.3 | -2.9045 | -7.3438 to 1.5347 | 0.1997 |
|  | Paule-Mandel | 2 | 162 | 145 | < 0.0001 | 99.3 | -2.9045 | -7.3438 to 1.5347 | 0.1997 |
|  | Maximum-likelihood | 2 | 162 | 145 | < 0.0001 | 99.3 | -2.9035 | -6.0425 to 0.2355 | 0.0698 |
|  | Hunter-Schmidt | 2 | 162 | 145 | < 0.0001 | 99.3 | -2.9035 | -6.0356 to 0.2286 | 0.0692 |
|  | Sidik-Jonkman | 2 | 162 | 145 | < 0.0001 | 99.3 | -2.9045 | -7.3293 to 1.5203 | 0.1982 |
|  | Hedges | 2 | 162 | 145 | < 0.0001 | 99.3 | -2.9045 | -7.3438 to 1.5347 | 0.1997 |
|  | Empirical Bayes | 2 | 162 | 145 | < 0.0001 | 99.3 | -2.9045 | -7.3438 to 1.5347 | 0.1997 |
|  | Excluded high risk of bias | 1 | 33 | 33 | - | - | - | - | - |

## **Supplementary Table 4**

| **Outcome** | **Analytic method** | **Number of studies** | **Number of animals/participants** | | **Heterogeneity** | | **Effect estimates** | | |
| --- | --- | --- | --- | --- | --- | --- | --- | --- | --- |
|  |  |  | **Intervention** | **Control** | **Chi^2^** | **I^2^ (%)** | **Standardized Mean Difference** | **95% CI** | **p-value** |
| *Animal* | |  |  |  |  |  |  |  |  |
| Rubbing | Overall | 5 | 77 | 37 | < 0.0001 | 88.1 | -2.2013 | -3.8027 to -0.5998 | 0.0071 |
|  | Oral administration | 5 | 77 | 37 | < 0.0001 | 88.1 | -2.2013 | -3.8027 to -0.5998 | 0.0071 |
|  | Sublingual administration | - | - | - | - | - | - | - | - |
| Sneezing | Overall | 5 | 53 | 33 | 0.0004 | 80.6 | -2.3844 | -3.6940 to -1.0747 | 0.0004 |
|  | Oral administration | 5 | 53 | 33 | 0.0004 | 80.6 | -2.3844 | -3.6940 to -1.0747 | 0.0004 |
|  | Sublingual administration | - | - | - | - | - | - | - | - |
| IL-4 | Overall | 4 | 82 | 44 | 0.0308 | 66.3 | -0.9416 | -1.8130 to -0.0702 | 0.0342 |
|  | Oral administration | 2 | 18 | 12 | 0.5614 | 0.0 | -1.8597 | -2.7794 to -0.9400 | < 0.0001 |
|  | Sublingual administration | 2 | 64 | 32 | 0.3440 | 0.0 | -0.4263 | -0.8562 to 0.0036 | 0.0519 |
| IgE | Overall | 4 | 64 | 30 | 0.0081 | 74.6 | -1.0498 | -2.2089 to 0.1094 | 0.0759 |
|  | Oral administration | 3 | 48 | 22 | 0.0137 | 76.7 | -1.4565 | -2.8456 to -0.0673 | 0.0399 |
|  | Sublingual administration | 1 | 16 | 8 | - | - | - | - | - |
| Eosinophil cell count | Overall | 3 | 78 | 39 | < 0.0001 | 89.9 | -1.8984 | -4.6783 to 0.8815 | 0.1807 |
|  | Oral administration | 1 | 14 | 7 | - | - | - | - | - |
|  | Sublingual administration | 2 | 64 | 32 | 0.3947 | 0.0 | -0.6454 | -1.0810 to -0.2099 | 0.0037 |

**Supplementary Table 5**

| **Outcome** | **Initial quality of evidence** | **Risk of bias** | **Inconsistency** | **Indirectness** | **Imprecision** | **Publication bias** | **Other considerations** | **Final quality of evidence** | **Importance** |
| --- | --- | --- | --- | --- | --- | --- | --- | --- | --- |
| Itching | High (RCTs)  ⊕⊕⊕⊕ | Not serious  (0)^a^  (High risk of bias ≤ 50% of the overall evidence) | Very serious  (-2)^b^  (I^2^ = 99.2%) | Not serious (0)^c^  (Eligible criteria were directness ) | Not Serious  (0)^d^  (SMD = -2.2564 , 95%CI = -5.9677 to 1.4548, p-value = 0.2334) | Not serious  (0)^e^ | No | Low  ⊕⊕⭘⭘ | Important |
| Nasal congestion |  | Not serious  (0)^a^  (High risk of bias ≤ 50% of the overall evidence) | Very serious  (-2)^b^  (I^2^ = 99.0%) |  | Not Serious  (0)^d^  (SMD = -1.8341, 95%CI = -5.0353 to 1.3671, p-value = 0.2615) | Not serious  (0)^e^ | No | Low  ⊕⊕⭘⭘ | Important |
| Runny nose |  | Not serious  (0)^a^  (High risk of bias ≤ 50% of the overall evidence) | Very serious  (-2)^b^  (I^2^ = 99.5%) |  | Not Serious  (0)^d^  (SMD = -2.8512, 95%CI = -7.9418 to 2.2395, p-value = 0.2723) | Not serious  (0)^e^ | No | Low  ⊕⊕⭘⭘ | Important |
| Sneezing |  | Not serious  (0)^a^  (High risk of bias ≤ 50% of the overall evidence) | Very serious  (-2)^b^  (I^2^ = 99.3%) |  | Not Serious  (0)^d^  (SMD = -2.9045, 95%CI = -7.3438 to 1.5347, p-value = 0.1997) | Not serious  (0)^e^ | No | Low  ⊕⊕⭘⭘ | Important |

**Note:** ^a^Not downgraded; ≤ 50% of studies were at high risk of bias

^b^Downgraded two levels due to very high heterogeneity (I² > 90%)

^c^Not downgraded; population, intervention, comparator, and outcomes were directly relevant

^d^Not downgraded; despite wide 95% CIs crossing the line of no effect, SMDs were consistently in the same direction across nasal symptom outcomes

^e^Not downgraded; could not be formally assessed due to the small number of studies

**Supplementary Figure 1**

**Supplementary Figure 2**

**Animal Rubbing**

**Supplementary Figure 3**

**Animal Sneezing**

**Supplementary Figure 4**

**Animal IL-4**

**Supplementary Figure 5**

**Animal IgE**

**Supplementary Figure 6**

**Animal Eosinophil cell count**

**Supplementary Figure 7**

**Human Itching**

**Supplementary Figure 8**

**Human Nasal congestion**

**Supplementary Figure 9**

**Human Runny nose**

**Supplementary Figure 10**

**Human Sneezing**

**Supplementary Figure 11**

**Animal Rubbing**

**Supplementary Figure 12**

**Animal Sneezing**

**Supplementary Figure 13**
